# PAC1 Receptor Knockout Mice Reveal Critical Links Between ER Stress, Myelin Homeostasis, and Neurodegeneration

**DOI:** 10.3390/ijms26178668

**Published:** 2025-09-05

**Authors:** Minduli Withana, Laura Bradfield, Margo I. Jansen, Giuseppe Musumeci, James A. Waschek, Alessandro Castorina

**Affiliations:** 1 Laboratory of Cellular and Molecular Neuroscience (LCMN), School of Life Sciences, Faculty of Science, University of Technology Sydney, Sydney, NSW 2007, Australia; umayaminduli.withana@student.uts.edu.au (M.W.); margo.jansen@student.uts.edu.au (M.I.J.); 2 Brain and Behaviour Group, School of Life Sciences, Faculty of Science, University of Technology Sydney, Sydney, NSW 2007, Australia; laura.bradfield@uts.edu.au; 3 School of Psychology, Faculty of Science, University of Sydney, Sydney, NSW 2006, Australia; 4 Department of Biomedical and Biotechnological Sciences, Section of Anatomy, Histology and Movement Science, School of Medicine, University of Catania, 95124 Catania, Italy; g.musumeci@unict.it; 5 Intellectual Development and Disabilities Research Centre, Semel Institute for Neuroscience and Human Behaviour/Neuropsychiatric Institute, David Geffen School of Medicine, University of California-Los Angeles, Los Angeles, CA 90095, USA; jwaschek@mednet.ucla.edu

**Keywords:** PAC1 receptor, endoplasmic reticulum (ER) stress 2, unfolded protein response (UPR), multiple sclerosis, myelin homeostasis, neurodegeneration, oligodendrocytes

## Abstract

The pituitary adenylate cyclase-activating polypeptide receptor 1 (PAC1) plays a pivotal role in central nervous system development and homeostasis. Comparisons of PAC1 knockout (PAC1^−/−^), heterozygous (PAC1^+/−^) and wild-type (PAC1^+/+^) mice demonstrate that PAC1 deficiency severely impairs pre-weaning survival and results in marked developmental deficits, including reduced postnatal weight and altered locomotor behavior. PAC1^−/−^ mice exhibited hyperlocomotion, reduced anxiety-like behavior, and transient deficits in motor coordination. Gene expression analyses revealed widespread dysregulation of oligodendrocyte-associated markers, with significant myelin reduction and decreased mature oligodendrocyte density in the corpus callosum. ER stress was evidenced in both white matter and motor cortex, as indicated by altered expression of UPR-related genes and increased phosphorylated (p)IRE1^+^ neurons. Retinal morphology was compromised in PAC1^−/−^ animals, with reduced overall retinal and ganglion cell layer thickness. Notably, no gross morphological or molecular abnormalities were detected in the spinal cord regarding myelin content or MBP expression; however, synaptic marker expression was selectively reduced in the ventral horn of PAC1-deficient mice. Together, these findings highlight a critical role for PAC1 in oligodendrocyte maturation, retinal development, and synaptogenesis, providing new insights with relevance in multiple sclerosis and other neurodevelopmental and neurodegenerative conditions.

## 1. Introduction

Multiple sclerosis (MS) is an immune-mediated demyelinating disorder of the central nervous system (CNS) that primarily affects young adults [1]. The disease is characterised by ongoing demyelination, activation of resident glia and infiltration of peripheral immune cells, leading to neurodegeneration [2].

MS manifests with different clinical courses, the most common being relapsing-remitting MS (RRMS). In RRMS, periods of symptom flare-ups (relapses) are followed by intervals of partial or complete recovery (remissions) before the next episode occurs [3]. Primary progressive MS (PPMS) and secondary progressive MS (SPMS) are also characterized by myelin loss and inflammation, although the inflammatory activity tends to decrease over time in these progressive forms. However, in these severe forms of MS, disease progression occurs more steadily without clear remissions, often leading to a faster and more pronounced CNS deterioration compared to RRMS [4].

Although the exact pathogenesis of MS remains elusive, it is well-established that the disease initiates with the aberrant activation of immune cells, including lymphocytes (T-helper, CD8+ cytotoxic, and B cells) and macrophages [5,6]. This misguided immune response leads to a devastating attack by pro-inflammatory cytokines against myelin antigens, ultimately resulting in axonal damage, gliosis, and the formation of CNS lesions (plaques), which are the typical imaging findings in MS patients [7,8]. Treatments for MS entail the longstanding use of immunosuppressive or immunomodulatory therapies, designed to reduce the abnormal activation of the immune system, thereby alleviating symptoms [9]. However, these therapies do not offer a cure and require lifelong management, often accompanied by significant side effects [10].

The endoplasmic reticulum (ER) is the principal subcellular structure responsible for the folding and maturation of proteins across all eukaryotic cells [11]. The ER is responsible for the folding of transmembrane and secreted proteins [12]. Transmembrane proteins such as the Inositol-Requiring Enzyme 1 (IRE1), Protein Kinase RNA-like Endoplasmic Reticulum Kinase (PERK) and Activating Transcription Factor 6 (ATF6) each operate as cellular detectors of stress by gauging the levels of unfolded proteins within the organelle [13,14]. Upon sensing ER stress, these proteins rapidly activate their own distinct arm of the unfolded protein response (UPR)—an evolutionary-conserved set of adaptive responses to restore cellular homeostasis—in the effort to dampen protein transcription and translation and attenuate protein synthesis [15]. This is underscored by the elevation of UPR markers such as the chaperone/pro-survival protein Binding immunoglobulin Protein (BiP, also known as Glucose-regulated protein 78 [GRP78]), as well as the same ER stress sensors PERK, IRE1 and ATF6 [16,17]. Noteworthy, whether UPR activation culminates in the promotion of cell survival or triggers apoptosis depends on the duration and severity of the ER stress stimulus [17], the balance between pro-survival and pro-apoptotic signals [18] and the balance of transcriptional outputs [19]. For example, activation of the pro-survival gene BiP favors survival, while increased expression of the pro-apoptotic factor C/EBP homologous protein (CHOP) (encoded by the gene *DDIT3*) can lead to the activation of cell death pathways [13,20]. In recent years, the significance of ER stress in neurodegenerative disease has gained more attention [21]. Analyses of brains from human MS patients have consistently shown augmented levels of ER stress markers such as BiP, XBP1 and CHOP in demyelinating lesions compared to healthy white matter or non-MS brain, establishing the relevance of this pathway in the death of myelin-producing oligodendrocytes and inflammatory responses of immune-cells within sites of degeneration [22]. In particular, the exacerbated expression of CHOP is considered a hallmark of active demyelination [22]. Most importantly, the causative factors that greatly impact the severity of ER stress and thereby myelin loss, remain unknown. Moreover, the endogenous elements that are activated to counter this stressful insult and reestablish homeostasis by directly/indirectly redirecting the UPR towards survival rather than apoptosis, remain elusive, which stands as the focal point of our study [23].

Pituitary adenylate cyclase activating peptide (PACAP) and vasoactive intestinal peptide (VIP) are two neuropeptides that are abundantly expressed in the CNS [24,25]. These neuropeptides exert protective and immunomodulatory effects through three G-protein-coupled receptors: the VPAC1 and VPAC2 receptors (collectively known as VPAC-type receptors), and the PAC1 receptor [26]. While PACAP and VIP bind to VPAC1 and VPAC2 receptors with similar high affinity, PACAP exhibits greater affinity (100- to 1000-fold) for the PAC1 receptor compared to VIP [27,28].

PACAP plays a critical role in neuroprotection [29] and cell regeneration [30], making it of considerable interest in the context of MS [28]. As highlighted in prior work, PACAP levels are significantly reduced in the cerebrospinal fluid (CSF) of individuals with MS [31], and the administration of PACAP in mouse murine models of MS leads to a marked alleviation of pathological symptoms [32,33]. In a previous review, we have proposed a potential link of the PACAP/PAC1 signalling axis and ER stress, highlighting the need for further investigation into this relationship [34]. Despite these insights, the direct impact of PAC1 receptor gene (aka *Adcyap1r1*, but from here on defined as PAC1) deletion on CNS myelination under physiological conditions remains unexplored.

In addition to its role in the brain and spinal cord, PAC1 is also highly expressed in the retina, an anatomical extension of the CNS composed of nine distinct layers, including a retinal ganglionic cell layer that houses CNS axons [35]. PAC1 receptors are prominently localized in several retinal layers in both rodents and humans, particularly in the ganglionic cell layer, inner plexiform layer, and inner nuclear layer. In these regions, PAC1 signalling is thought to influence visual signal processing and provide neuroprotection in response to oxidative stress or injury [36,37,38]. PAC1 activity has also been implicated in the regulation of retinal cell survival and circadian rhythm entrainment via its effects on both neuronal and glial cell populations [39,40,41]. Nonetheless, the structural and functional consequences of PAC1 receptor deficiency in the retina have not been systematically examined in PAC1 knockout models, leaving a critical gap in our understanding of its role in retinal physiology and broader CNS-related neurodegenerative processes.

In this study, we used mice that are globally deficient for the PAC1 gene (PAC1^−/−^ mice) as well as heterozygous PAC1 knockouts (PAC1^+/−^ mice) and wild-types (PAC1^+/+^ mice) to investigate the behavioural, histological and molecular phenotypic changes triggered by global neuropeptide receptor ablation in myelin health, myelin cell survival, endogenous activation of ER stress responses, spinal cord and eye.

## 2. Results

### 2.1. Complete Loss of PAC1 Elevates the Percentage of Pre-Weaning Deaths

**Figure 1:**
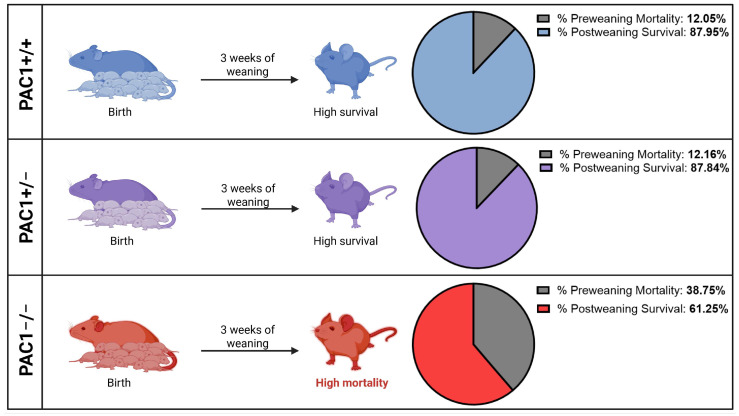
Preweaning deaths and post-weaning survival rates in PAC1^+/+^ (top panel), PAC1^+/−^ (middle panel) and PAC1^−/−^ mice (bottom panel). Data shown in each pie chart indicates the percentage of mice that succumbed preweaning over the total mice bred for that specific genotype. Total number of mice born per each genotype from January until December 2024 was: PAC1^+/+^ mice = 83 (preweaning deaths = 10), PAC1^+/−^ mice = 74 (preweaning deaths = 9), and PAC1^−/−^ mice = 80 (preweaning deaths = 31).

For this study, colonies of mutant mice and wild types (PAC1^+/+^) were bred in house. Pups were weaned at 3 weeks of age. Interestingly, PAC1^−/−^ pups consistently displayed stunted weight and a runt-like phenotype in comparison to both PAC1^+/+^ and PAC1^+/−^ animals. Furthermore, and perhaps more significantly, PAC1^−/−^ pups displayed extremely poor survivability in the first 3 weeks of life, with 38.75% of all knockout animals birthed failing to surpass the initial preweaning period, as opposed to only 12.05% in PAC1^+/+^ and 12.16% amongst PAC1^+/−^ groups (Figure 1). Overall, our findings demonstrate that PAC1^−/−^ mice show a ~3-fold higher preweaning mortality rate compared to wild types (PAC1^+/+^), although survivability was alike across all genotypes after weaning.

### 2.2. PAC1 Deletion Alters Locomotor Activity and Anxiety-Related Behaviours

**Figure 2:**
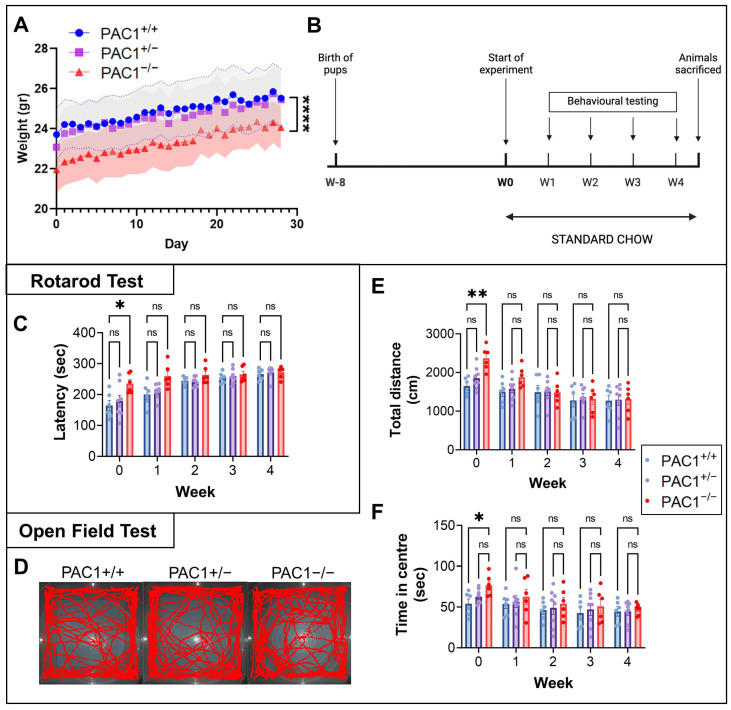
Effects of partial and complete PAC1 deletion on body weight, balance, coordination, and locomotion. (**A**) Average daily weights ± SEM of PAC1^+/+^, PAC1^+/−^ and PAC1^−/−^ mice during the testing period (4 weeks). **** *p* < 0.0001 vs. PAC1^+/+^ or PAC1^+/−^ mice, as computed using repeated measures ANOVA and Tukey post hoc analysis. (**B**) Schematic of the experimental timeline, starting at birth of the pups, followed by the onset of behavioural assessments at 8 weeks of age, and concluding with tissue collection at 12 weeks. (**C**) Latency to fall of mice undergoing weekly Rotarod tests. Results shown are the mean latency to fall (in seconds) ± SEM of PAC1^+/+^ (*n* = 6), PAC1^+/−^ (*n* = 8) and PAC1^−/−^ (*n* = 6) mice. (**D**) Representative line track and (**E**) total distance travelled by mice of each genotype in the Open Field arena each week (over a 3 min testing period), and (**F**) time spent in centre. * *p* < 0.05 or ** *p* < 0.01 vs. PAC1^+/+^ mice, as determined using a linear mixed-effects model using genotype and time as fixed effects and mouse identification as a random effect. Post hoc comparisons were corrected using Tukey method. ns = not significant.

Experiments to investigate locomotor behaviors commenced when mice of each genotype (PAC1^+/+^, PAC1^+/−^ and PAC1^−/−^) were 8 weeks old. Mice were weighed daily over a four-week period and tested weekly for changes in balance, locomotion, and anxiety (Figure 2A,B). Interestingly, although PAC1^−/−^ mice consistently displayed reduced survival at birth and were significantly smaller in size (**** *p* < 0.0001 vs. PAC1^+/+^ or PAC1^+/−^ mice; Figure 2A), although their postweaning growth rates were comparable to those of heterozygous and wild-type littermates. In contrast, PAC1^+/−^ mice maintained a weight similar to that of wild-type (PAC1^+/+^) animals throughout the experimental period.

Using the Rotarod test, we assessed the effect of PAC1 deletion on balance and coordination by measuring the average latency to fall from an accelerating rod (Figure 2C). At baseline, PAC1^−/−^ but not PAC1^+/−^ mice showed increased latency to fall (* *p* < 0.05 vs. PAC1^+/+^ mice; Figure 2C). Although latency remained slightly elevated in homozygous knockouts after 1 week, the difference was no longer statistically significant in subsequent testing sessions.

The Open Field Test was used to assess general locomotion and anxiety-like behaviour following pan- or partial ablation of the PAC1 gene. Representative trajectory plots of each group’s exploratory behaviour in the arena are shown in Figure 2D. Consistent with Rotarod findings and previous reports [42], PAC1^−/−^ mice exhibited increased locomotion during the initial session (** *p* < 0.01 vs. PAC1^+/+^ mice at Week 0; Figure 2E), an effect not observed in PAC1^+/−^ mice. This was accompanied by increased time spent in the centre of the arena by PAC1^−/−^ mice during the first session only (* *p* < 0.05 vs. PAC1^+/+^ mice at Week 0; Figure 2F). These differences were not maintained in later sessions.

### 2.3. PAC1 Receptor Deletion Disrupts Oligodendrocyte-Associated Gene Expression and Reduces Mature Oligodendrocyte Density

**Figure 3:**
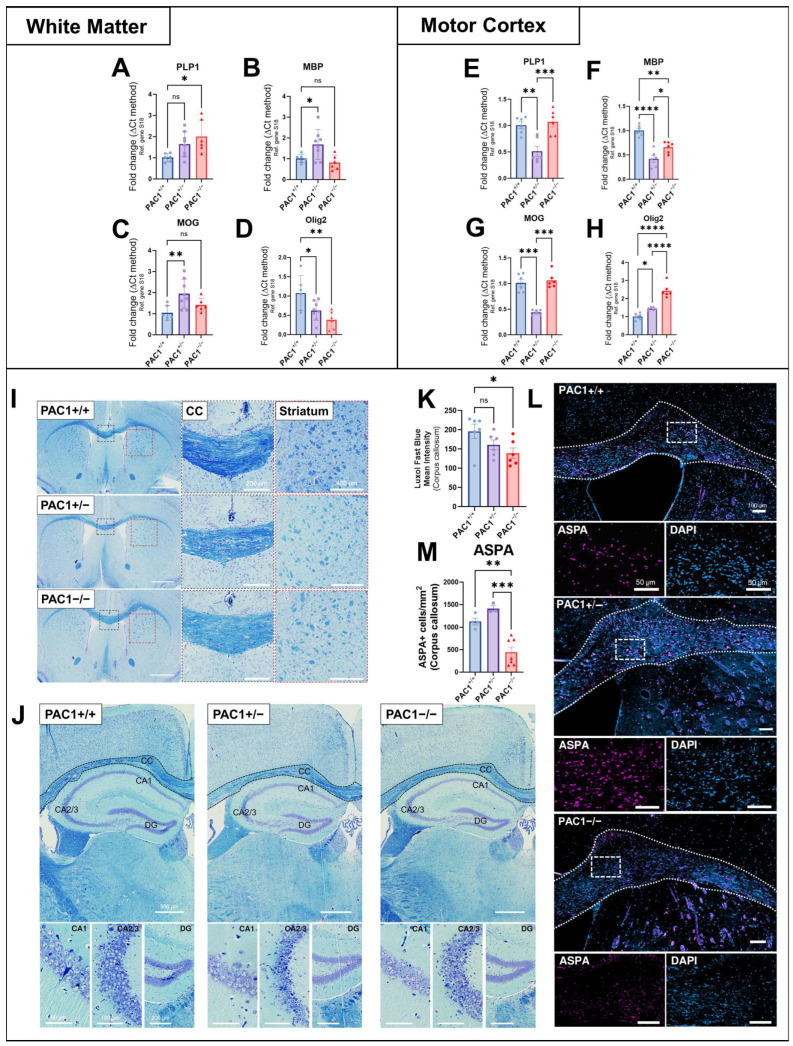
Effects of global PAC1 deficiency on the expression of myelin-associated genes, myelin and mature oligodendrocyte density. Analyses of PLP (**A**,**E**), MBP (**B**,**F**), MOG (**C**,**G**) and Olig2 (**D**,**H**) gene expression in the CNS white matter and motor cortex, respectively, of PAC1^+/+^, PAC1^+/−^ and PAC1^−/−^ mice. Results are presented as the mean fold change ± SEM from *n* = 6–8 mice per genotype. Representative Luxol Fast Blue (LFB) staining (with insets at higher magnification), showing myelin density in the corpus callosum (**I**) and hippocampus/cortex (**J**) of mutant mice. Quantification of the mean myelin intensity ± SEM (*n* = 6 mice per group) in the corpus callosum of mice, performed via LFB staining (**K**). Representative immunofluorescence staining of ASPA+ cells (with insets at higher magnification) of the corpus callosum of wild-type (PAC1^+/+^), heterozygous (PAC1^+/−^) and homozygous PAC1 knockout mice (PAC1^−/−^) (**L**). Stereology of the average ASPA^+^ cell density (cells/mm^2^) ± SEM (*n* = 3–7 mice per group) (**M**). * *p* < 0.05, ** *p* < 0.01, *** *p* < 0.001 or **** *p* < 0.0001 vs. PAC1^−/−^ (or PAC1^+/−^, as indicated) after analysis using One-Way ANOVA and Tukey’s multiple comparisons tests. ns = not significant.

To investigate the role of PAC1 signalling in myelin-associated gene expression, we quantified transcript levels of key oligodendrocyte lineage and myelin genes in the callosal and cortical (motor cortex) white matter of PAC1^+/+^, PAC1^+/−^ and PAC1^−/−^ mice. In mice with homozygous PAC1 deletion (PAC1^−/−^), we observed a significant increase in mRNA expression of PLP1, encoding a key structural component of CNS myelin compared to wild-types (* *p* < 0.05 vs. PAC1^+/+^ mice; Figure 3A). However, Olig2 expression, a transcription factor essential for early oligodendrocyte development, was significantly reduced in these same animals (** *p* = 0.0027; Figure 3D). MBP and MOG transcript levels remained unchanged in PAC1^−/−^ mice (Figure 3B,C).

Conversely, PAC1^+/−^ mice showed a significant upregulation of both MBP (* *p* < 0.05; Figure 3B) and MOG mRNAs (** *p* < 0.01; Figure 3C), while Olig2 expression was similarly reduced as in homozygous knockouts (* *p* < 0.05; Figure 3D). Interestingly, PAC1^+/−^ and PAC1^−/−^ mice displayed opposing changes in PLP1, MBP, and MOG expression, suggesting that partial versus complete loss of PAC1 signalling may differentially affect myelin gene regulation. This could reflect compensatory or threshold-dependent mechanisms, where partial loss of PAC1 signalling promotes adaptive myelin gene upregulation, while complete ablation disrupts regulatory networks governing oligodendrocyte maturation.

Analysis of the same myelin-associated genes in the motor cortex revealed a different pattern of transcript dysregulation in PAC1^−/−^ mice. Specifically, MBP transcripts were significantly reduced with respect to wild-type mice (** *p* < 0.01; Figure 3F), whereas Olig2 expression was significantly increased (**** *p* < 0.0001; Figure 3H). PLP1 and MOG were unchanged in the motor cortex of PAC1^−/−^ mice (Figure 3E,G).

In PAC1^+/−^ mice, both PLP1, MBP and MOG transcript levels were significantly reduced (** *p* < 0.01, **** *p* < 0.0001 and *** *p* < 0.001, respectively; Figure 3E–G), while Olig2 expression was increased (* *p* < 0.05; Figure 3H).

Luxol Fast Blue (LFB) staining, used to evaluate myelin content, revealed a statistically significant reduction in myelin density in the corpus callosum of PAC1^−/−^ mice (* *p* < 0.05), with only a slight decrease in PAC1^+/−^ lines (Figure 3I,K). In the striatum, we also observed some LFB discoloration in both PAC1^+/−^ and, more prominently, in PAC1^−/−^ mice (Figure 3I); however, we were not able to quantify LFB intensity due to the inherent heterogeneity of the tissue in this region. Additionally, qualitative inspection of the hippocampus and cortex suggested modestly reduced myelin staining in both PAC1^+/−^ and PAC1^−/−^ mice (Figure 3J), though this was not quantitatively assessed for the same reasons outlined above.

Immunohistochemical analysis using aspartoacylase (ASPA), a marker of mature oligodendrocytes, revealed a reduction in ASPA^+^ cell density in the corpus callosum of PAC1^−/−^ mice (** *p* < 0.01; Figure 3M), but not in PAC1^+/−^ mice, suggesting that an overall decrease in mature oligodendrocyte populations can be appreciated only when PAC1 is completely inactivated.

### 2.4. Effects of Global PAC1 Deletion on the Expression of ER Stress Markers in the White Matter and Motor Cortex

**Figure 4:**
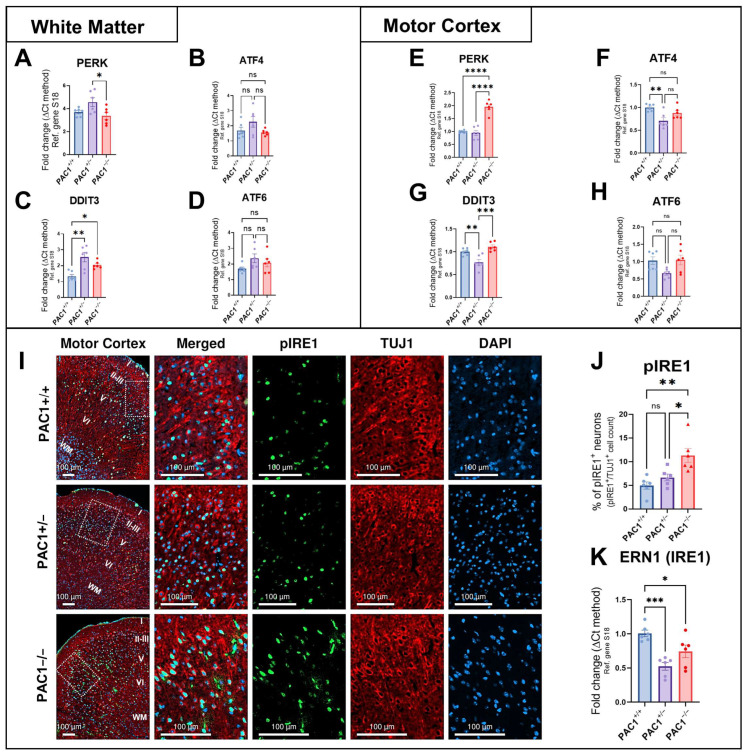
Effects of partial and complete PAC1 gene deletion on UPR activation. Gene expression of (**A**,**E**) *PERK*, (**B**,**F**) *ATF4*, (**C**,**G**) *DDIT3* and (**D**,**H**) *ATF6* in the white matter and motor cortex of PAC1^+/+^, PAC1^+/−^ and PAC1^−/−^ mice. Results presented are the mean fold change ± SEM (*n* = 6 mice per genotype). (**I**) Representative photomicrographs (with insets at higher magnification taken from the dashed white areas) of the motor cortex (approximate boundaries of cortical layers I–VI are indicated) that have been co-stained with pIRE1 and TUJ1 and counterstained with the nuclear dye DAPI and (**J**) related stereological measures of the ratio of pIRE1^+^ neurons (TUJ1^+^ cells) in each region of interest (each ROI = 40,000 µm^2^, *n* = 6 mice). Data shown is the average percentage of pIRE1+/TUJ1+ cells per ROI ± SEM from 6 mice per group. (**K**) Real-time qPCR analysis of *ERN1* gene expression in the motor cortex of mutant mice. Each data point is the mean ± SEM (*n* = 6 mice). * *p* < 0.05, ** *p* < 0.01, *** *p* < 0.001 and **** *p* < 0.0001 vs. PAC1^+/+^ (or PAC1^+/−^, as shown), as determined using One-Way ANOVA followed by Tukey’s multiple comparisons test. ns = not significant.

Considering the stark variation in myelin markers discovered, we sought to elucidate if the deletion of PAC1 from these animals compromised cellular homeostasis and caused ER stress within the same brain regions. To address this, we began by examining UPR the expression levels of key UPR genes in the white matter, the primary site of demyelination during MS (Figure 4A). Expression of PERK (also known as EIF2AK3), a key upstream activator of one of the three major UPR signalling pathways, was downregulated in PAC1^−/−^ mice in comparison to heterozygous mice but not wild-types (* *p* = 0.0293, PAC1^+/−^ mice; Figure 4A). No changes in ATF4 mRNAs were observed across genotypes (*p* > 0.05; Figure 4B). Expression levels of DDIT3 were increased in the white matter of both PAC1^+/−^ (* *p* = 0.0013 vs. PAC1^+/+^ mice) and PAC1^−/−^ mice (* *p* = 0.0471; Figure 4C). ATF6 gene expression levels were only marginally elevated in PAC1^+/−^ and PAC1^−/−^ mice, although not at statistically significant levels (*p* > 0.05; Figure 4D).

To determine whether PAC1 gene ablation interfered with ER stress responses in a neuron-rich region such as the motor cortex, we interrogated the same set of genes as in the white matter. PERK gene expression spiked in the PAC1^−/−^ motor cortex compared to both wild-type and heterozygous groups (**** *p* < 0.0001 vs. PAC1^+/+^ mice for both; Figure 4E). ATF4 transcript levels were unchanged in PAC1^−/−^ mice (*p* > 0.05; Figure 4F), and so were DDIT3 (Figure 4G) and ATF6 transcripts (Figure 4H).

In PAC1^+/−^ animals, PERK gene expression was unaffected (Figure 4E), whereas ATF4 and DDIT3 expression levels were significantly reduced (** *p* < 0.01 vs. PAC1^+/+^ mice for both; Figure 4F,G). ATF6 expression was not affected in heterozygous mice (Figure 4H).

To further investigate if the disruptions of the motor cortex UPR were more prevalent in cortical neurons, we performed co-localisation studies using the pan-neuronal marker TUJ1 (also known as βIII-tubulin) and IRE1 (phosphorylated at Ser 724; pIRE1), which is essential for the activation of the UPR under ER stress [43]. Stereological measures revealed significantly increased percentage of pIRE1^+^ neurons in the motor cortex of both PAC1^−/−^ and heterozygous animals (** *p* = 0.0017 and * *p* = 0.0174 vs. PAC1^+/+^ mice, respectively; Figure 4I,J), suggesting that PAC1 gene ablation promotes neuronal ER stress engagement. In contrast, transcript levels of ERN1 (which encodes for IRE1 protein) were significantly reduced in PAC1^−/−^ (* *p* = 0.0406 vs. PAC1^+/+^ mice) and more prominently in PAC1^+/−^ mice (*** *p* = 0.0005; Figure 4K). This apparent de-coupling between increased pIRE1^+^ neurons and reduced ERN1 transcript levels may reflect differences in protein turnover or RNA instability, as gene expression changes do not necessarily parallel phospho-protein dynamics.

### 2.5. PAC1 Deletion Reduces the Thickness of Both the Retinal Ganglion Cell Layer and the Overall Retina

**Figure 5:**
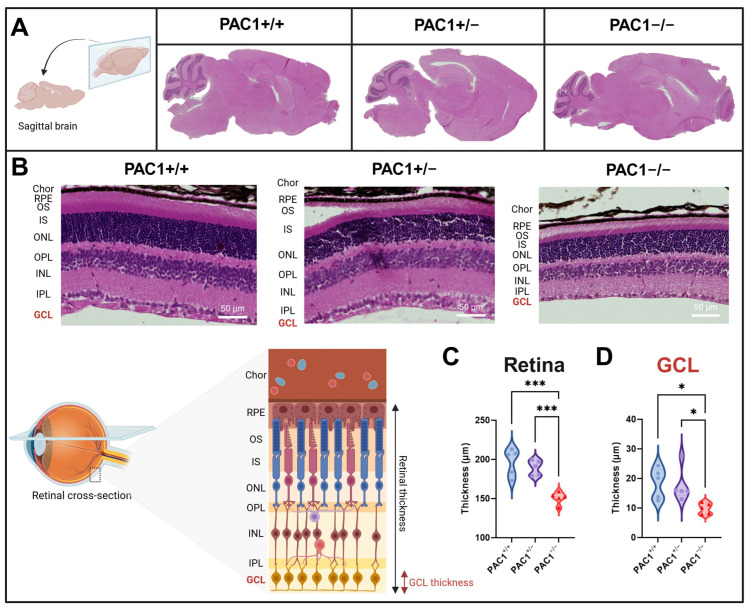
Gross morphology and morphometrical examination of the retina in PAC1^−/−^ and PAC1^+/−^ mice. Representative brain sagittal sections of PAC1^+/+^, PAC1^+/−^ and PAC1^−/−^ mice (**A**) and retinal cross sections depicting the different layers after staining using H&E (**B**). Individual layers are labelled as follows (from top to bottom): Chor = choroid, RPE = Retinal pigmented epithelium OS/IS = Outer/Inner segment of the Rode and Cone layer, ONL = Outer Nuclear Layer, OPL = Outer Plexiform Layer, INL = Inner Nuclear Layer, IPL = Inner Plexiform Layer, GCL = Ganglionic Cell Layer. Violin plots showing the average retinal thickness ± SEM (*n* = 5 mice per group) (**C**) and that of the GCL (*n* = 5) (**D**). * *p* < 0.05 or *** *p* < 0.001 vs. PAC1^−/−^ or PAC1^+/−^ mice, as calculated using One-Way ANOVA followed by Tukey’s multiple comparisons test. Scale bar = 50 µm.

Given the ubiquitous expression of the PAC1 receptor in the CNS [44] and retina [37], as well as recent finding pointing to a critical homeostatic role of the receptor in retinal function in a preclinical model of MS [41], we sought to determine the effect of ubiquitous PAC1 ablation on the gross brain architecture of the brain and retina using H&E staining. As shown in Figure 5A, examination of sagittal sections of the brain revealed no gross morphological changes among genotypes. In contrast, analysis of retinal cross sections (Figure 5B) revealed a marked reduction in the retinal thickness in PAC1^−/−^ mice both in comparison to PAC1^+/−^ or PAC1^+/+^ mice (*** *p* < 0.0002 vs. PAC1^+/+^ mice and *** *p* < 0.005 vs. PAC1^+/−^ mice, respectively; Figure 5C). Furthermore, the thickness of the retinal ganglionic cell layer was also significantly diminished in knockout animals compared to the two other genotypes (* *p* < 0.05 vs. both PAC1^+/+^ and PAC1^+/−^ mice; Figure 5D), in line with prior findings [41].

### 2.6. PAC1 Ablation Causes No Changes in Spinal Cord Myelin Intensity and Gene Expression

**Figure 6:**
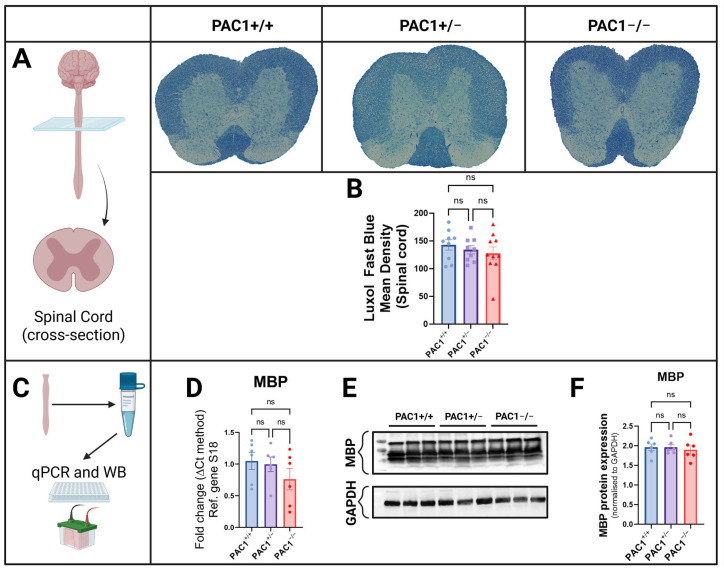
Myelin density and MBP expression in the spinal cord of PAC1^−/−^ mice. Representative LFB-stained cross sections of the spinal cord of PAC1^+/+^, PAC1^+/−^ and PAC1^−/−^ mice (**A**) and related quantifications of mean LFB density ± SEM (9–10 sections per group from *n* = 4–5 mice) (**B**). Real-time qPCR analyses of MBP gene expression in mutant mice. Results are the average fold change ± SEM (*n* = 6 mice per group) (**C**,**D**). Representative Western blot of MBP expression (**E**) and related densitometry across the different genotypes (*n* = 6 mice per group). Data shown is the mean ± SEM (**F**). ns = not significant. GAPDH = Glyceraldehyde-3-phosphate dehydrogenase.

In light of the recent evidence pointing to the importance of spinal PAC1 receptors in nociceptive responses [45], we harnessed our mutant mouse colonies to determine whether PAC1 gene inactivation interfered with myelin density in the spinal cord, as well as the expression of MBP, a major structural myelin protein. As shown (Figure 6A,B), LFB staining intensity did not vary significantly across genotypes. MBP gene expression was unaffected by PAC1 deletion (partial or complete), expect for a marginal and not statistically significant reduction in PAC1^−/−^ mice (Figure 6C,D). These results were corroborated by Western blots, showing that spinal MBP expression was not affected by PAC1 deletion (Figure 6E,F).

### 2.7. PAC1 Deficiency Alters Presynaptic Markers in the Spinal Cord

**Figure 7:**
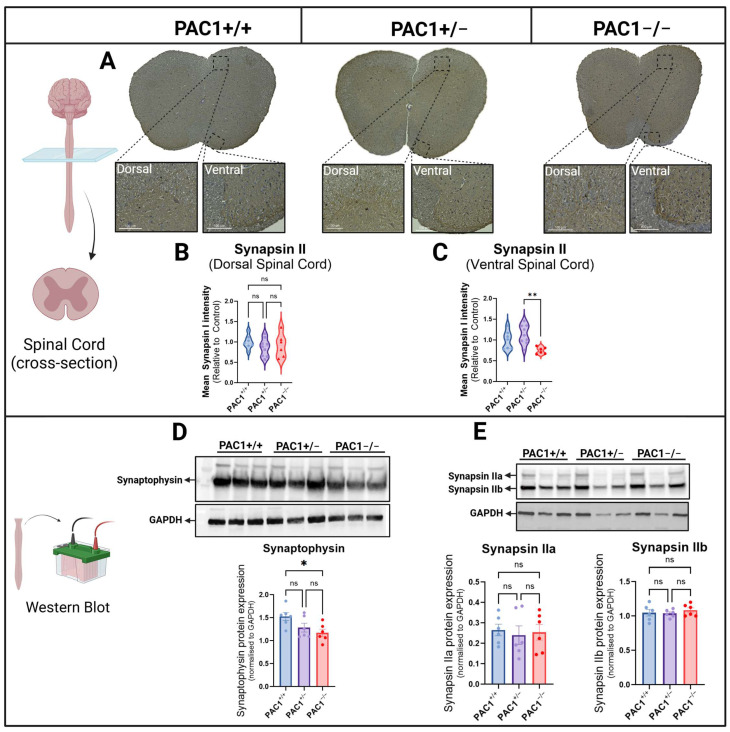
Expression of synapse-associated markers in the spinal cord of PAC1^+/+^, PAC1^+/−^ and PAC1^−/−^ mice. Representative Synapsin II immunoreactivity (IR) in spinal cord cross-sections from PAC1^+/+^, PAC1^+/−^ and PAC1^−/−^ mice (**A**) and related quantifications of the mean Synapsin II IR ± SEM (6–10 sections from *n* = 4–5 mice per genotype) from the dorsal (**B**) and ventral horns (**C**). Western blot showing Synaptophysin protein expression (**D**) and Synapsin II (showing both IIa and IIb isoforms) (**E**) and corresponding densitometric analyses using spinal cord lysates from mutant mice (*n* = 6 mice per genotype). Results shown in bar graphs are the mean ± SEM (**D**,**E**). * *p* < 0.05, ** *p* < 0.01 vs. PAC1^+/+^ mice, as determined by One-way ANOVA and Tukey post hoc test. ns = not significant.

Considering the abundance of PAC1 receptors in the spinal cord [45] and the fact that synapse dysfunction in this structure is a commonly observed pathological feature of MS [46,47], we were interested in determining if partial or complete deletion of the PAC1 receptor gene affected the expression of both Synapsin II and Synaptophysin, two prominent pre-synaptic vesicle-associated proteins critical for synaptic transmission and plasticity, with documented synaptic loss linked to disease progression in MS and preclinical models of disease [48,49]. For this purpose, we conducted Synapsin II immunohistochemistry in coronal sections of the spinal cord (Figure 7A). Analyses of immunoreactivity (IR) revealed abundant expression in grey matter, especially in the ventral horns (Figure 7A). Quantification of Synapsin II IR showed no changes in the dorsal horn among PAC1^+/+^, PAC1^+/−^ and PAC1^−/−^ mice (*p* > 0.05 vs. PAC1^+/+^ mice; Figure 7B), whereas a significant reduction was seen in the ventral horns of PAC1^−/−^ in comparison with heterozygous mice (** *p* < 0.005 vs. PAC1^+/−^ mice; Figure 7C).

To complement these findings, we analysed the protein expression of Synaptophysin, Synapsin IIa and IIb by Western blot (Figure 7D,E). As shown (Figure 7D), Synaptophysin expression was reduced in the spinal cord of PAC1^−/−^ mice (* *p* < 0.05 vs. PAC1^+/+^ mice), but not in PAC1^+/−^ mice (Figure 7D). Conversely, quantitative evaluation of both Synapsin IIa and IIb isoforms showed comparable results across genotypes, with no significant changes with respect to PAC1^+/+^ mice (Figure 7E).

## 3. Discussion

This study offers the first in vivo characterisation of the brain and retinal phenotype resulting from either heterozygous (PAC1^+/−^) or global deletion (PAC1^−/−^) of the PAC1 receptor. Our findings demonstrate that PAC1 is critical for maintaining myelin integrity, oligodendrocyte survival, and ER stress homeostasis under physiological conditions in the brain, and proper retina morphogenesis. The observed pre-weaning lethality, combined with subtle but region-specific neuroanatomical and molecular alterations, highlights the essential role of PAC1 across CNS compartments (Table 1).

Behavioural analyses showed that PAC1^−/−^ mice display increased baseline locomotion, enhanced balance and greater anxiety-like behaviours during the initial testing session compared to PAC1^+/+^ or PAC1^+/−^ animals, consistent with earlier reports [42]. However, these differences were not sustained in subsequent testing sessions. One possible explanation is behavioural habituation. PAC1^−/−^ mice may exhibit heightened response to novelty or stress during the first exposure due to altered neuropeptide signalling or dysregulation of stress-responsive circuits. Once the environment becomes familiar, their behavioural output normalises, masking the initial group differences. This aligns with the known role of PAC1 in mediating acute stress responses [50] and highlights a potential deficit in sensory or emotional regulation rather than sustained motor or affective dysfunction. An additional explanation is that compensatory mechanisms may mitigate behavioural deficits over time. Despite the constitutive loss of PAC1, other signalling pathways (e.g., VPAC2 or CRF receptor-mediated cascades) may adjust to preserve behavioural homeostasis. This is consistent with the broader capacity of the neuropeptide system for redundancy and plasticity.

The high rate of pre-weaning mortality among PAC1^−/−^ offspring (~3-fold increase) underscores a critical developmental role for PAC1 that may extend beyond the nervous system. PAC1-deficient pups also exhibited persistently reduced body weight, suggestive of a growth or metabolic deficiency, consistent with prior findings linking PACAP-PAC1 signalling to appetite regulation and energy homeostasis [51,52,53,54].

At the molecular level, PAC1 deletion caused a significant reduction in mature oligodendrocytes in the corpus callosum, as evidenced by decreased ASPA^+^ cell counts and altered expression of oligodendrocyte lineage markers. Olig2 expression was decreased, while PLP1 was paradoxically upregulated, possibly reflecting a compensatory response to impaired myelination. These findings are supported by previous in vitro and ex vivo studies demonstrating that PACAP promotes OPC proliferation and regulates myelin-cells maturation [55,56,57]. In contrast, in the motor cortex, Olig2 expression increased while MBP decreased, suggesting that although progenitor production may be elevated, differentiation into functional oligodendrocytes is compromised. This region-specific divergence in response may reflect varying thresholds of vulnerability to PAC1 loss across CNS areas. In addition, we observed qualitative evidence of myelin disruption outside of the corpus callosum. Specifically, LFB staining revealed modest discoloration in the striatum of both PAC1^+/−^ and, more prominently, PAC1^−/−^ mice, although quantification was not feasible due to tissue heterogeneity. Similarly, hippocampal and cortical sections suggested reduced myelin staining in knockout animals, again assessed qualitatively. While preliminary, these observations support a broader role for PAC1 in maintaining myelin integrity across multiple brain regions, warranting further quantitative investigation.

Consistent with our hypothesis that PAC1 helps buffer cells from ER stress, we observed dysregulation of key UPR markers. In the corpus callosum of PAC1^−/−^ mice, DDIT3 expression (which encodes for the protein CHOP) was elevated while PERK was suppressed, suggesting a pro-apoptotic shift in the stress response that may drive oligodendrocyte loss. In the motor cortex, upregulation of PERK, pIRE1 and DDIT3 indicates a sustained ER stress burden, likely representing an overwhelmed adaptive response. These results align with previous studies showing that DDIT3/CHOP elevation is associated with active demyelination and oligodendrocyte apoptosis in MS lesions [22], and with work in skeletal muscle showing similar ER stress marker induction upon PAC1 deletion [58]. At a mechanistic level, PAC1 receptor activation is known to engage multiple intracellular signalling pathways, including adenylate cyclase–cAMP and PLC–IP3 cascades [59]. The PLC pathway regulates IP3 receptor-mediated calcium release from the ER, which is critical for protein folding and ER homeostasis [60,61]. Loss of PAC1 signalling is therefore likely to disrupt ER calcium dynamics, leading to protein misfolding and maladaptive activation of the unfolded protein response (UPR) [34]. This provides a direct molecular link between PAC1 deletion and the elevated ER stress markers observed in our study.

Retinal analysis revealed significant thinning of the ganglion cell layer in PAC1^−/−^ mice, consistent with PAC1 known localisation in this layer and its proposed neuroprotective role in the retina [35,36,37,38]. This is particularly relevant given that retinal thinning is a hallmark of MS-related neurodegeneration and correlates with clinical disability [62]. In addition, prior work has shown that selective deletion of PAC1 in retinal neurons alone was sufficient to induce ganglion cell layer thinning and increased axonopathy under neuroinflammatory conditions [41]. In contrast, the current study utilised a global PAC1 knockout model, resulting in PAC1 loss across multiple CNS cell types, including neurons, astrocytes, oligodendrocytes, and microglia. This broader deletion may account for additional or divergent phenotypic changes observed in this study, particularly in white matter and cortical regions. These findings suggest that the effect of PAC1 signalling on CNS integrity is highly context- and cell type-dependent, with potentially synergistic or compensatory roles played by different CNS-resident populations.

For example, the differential regulation of myelin-associated genes in PAC1^+/−^ vs. PAC1^−/−^ mice could reflect non-linear or biphasic responses to partial versus complete receptor loss across cell types, such as astrocyte-mediated buffering of inflammatory or trophic signals. In this regard, the combined deletion across glial and neuronal compartments may reveal vulnerabilities or interactions not evident in neuron-specific models. Such distinctions have important implications for understanding how PAC1 exerts neuroprotective roles in health and disease.

Similarly, our spinal cord data showed that although myelin markers were largely unaffected at baseline, synaptic markers such as Synaptophysin and Synapsin II levels were reduced in knockouts. Given previous findings that synaptic loss is observed in the spinal cord of people with MS and the EAE model [46,63], these results raise the possibility that PAC1 loss could prime the spinal cord for synaptic vulnerability under disease conditions.

While our findings offer valuable new insight, several limitations must be noted. First, the use of global knockouts limits our ability to distinguish between cell-intrinsic and systemic effects of PAC1 deletion. This limitation is particularly relevant considering our earlier work targeting PAC1 only in retinal neurons, which produced distinct results that may reflect more restricted pathophysiological mechanisms. The broader deletion strategy used here may better model systemic CNS dysfunction but complicates mechanistic dissection. Second, we assessed baseline physiology without inducing a demyelinating challenge such as cuprizone or EAE, which may have unmasked additional deficits. However, in this regard, it should be noted that the scope of this work was to characterise key phenotypic alterations in the CNS of PAC1^−/−^ mice (as well as PAC1^+/−^ mice) to help unveil the importance of PAC1 receptors in CNS homeostasis and/or vulnerability to neurological conditions, including but not limited to MS. Furthermore, although we analysed ER stress markers, future work should incorporate functional assays to directly assess protein misfolding, calcium homeostasis, and apoptosis.

In summary, this study highlights the essential role of PAC1 in CNS homeostasis, particularly in supporting oligodendrocyte viability and limiting ER stress. By linking PAC1 deletion to impaired PLC–IP3 signalling and disruption of ER calcium balance, our findings provide a mechanistic rationale for the observed ER stress phenotype. The region-specific nature of the observed deficits suggests that certain CNS compartments may be more vulnerable to PAC1 loss than others. Future studies should investigate whether PAC1 activation can enhance remyelination or neuroprotection in demyelinating disease models, potentially advancing its relevance as a therapeutic target in MS and/or other demyelinating disorders.

## 4. Materials and Methods

### 4.1. Animals and Study Design

For this study, PAC1 knockout (PAC1^−/−^) mice originally generated on a mixed background (129/Sv × C57BL/6) by Jamen and collaborators [64] were kindly provided from collaborators at the Heart Research Institute (Dr. Melissa Farnham, Sydney, Australia). Upon arrival, as knockout females failed to display fertility [65], PAC1^+/−^ (heterozygous) females were crossbred with PAC1^−/−^ males over multiple generations to generate the desired number of knockout animals needed for the study [66]. Twenty PAC1^−/−^ (*n* = 6), PAC1^+/−^ (*n* = 8) and PAC1^+/+^ (*n* = 6) male and female mice were allowed to grow to 8 weeks old prior to the commencement of the study in the Ernst Animal Facility at the University of Technology Sydney. Mice were housed within independently EPA-filtered aerated cages (2 to 4 mice/cage) and allowed to acclimatize to the housing conditions for 1 week prior to the inception of the study. They were given ad libitum access to food (standard chow) and water and were exposed to a 12 h light/dark cycle. All breeding protocols (ETH21-6004) and experimental procedures (ETH21-6281) for this study were approved by the Animal Care and Ethics Committee (ACEC) at the University of Technology Sydney. Biosafety approval was obtained for the use of genetically modified organisms (ETH21-6743) and all protocols were conducted in accordance with the guidelines of the National Health and Medical Research Council of Australia. Open Field (OF) test and Rotarod behavioral assessments were performed on day 0 and every 7 days thereafter for 4 weeks. OF assessment was utilised to measure for changes in general locomotion and exploratory behaviour [67], as dampened motor activity in the OF, elevated atypical behaviour and thigmotaxis (movement towards solid structures) is often suggestive of aberrant neural pathways and atypical brain activity [68,69,70]. Likewise, the rotarod test was employed to assess motor coordination and balance in animals [71]. Animal weights were monitored every day as a measure of general health until the conclusion of the study on day 29. Mice were sacrificed by carbon dioxide asphyxiation via EA-33000TS SMARTBOX^®^ Prodigy (Biological Associates Pty. Ltd., Gladesville, NSW, Australia) at a rate of 3.0 L/min. After euthanasia, brains and spinal cords were collected and immediately snapped frozen for RNA studies or placed in 4% paraformaldehyde for immunohistochemical assessments. Eyes were extracted using tweezers and immersed in paraformaldehyde for 30 min, then perforated twice using a 23-gauge needle to release intraocular fluid pressure and allow the fixative to enter the posterior chamber of the eye to prevent collapse during processing.

### 4.2. Open Field Test

Mice were acclimatised to a darkened environment for 30 min. The OF box is an open area formed by a 40 cm (L) × 40 cm (W) × 50 cm (H) opaque Perspex box. Each mouse was placed at the centre of the box, and the movements of the animal were recorded for 5 min using a Sony FDRAX53 4K Full HD Handycam infrared video camera (JB Hi-Fi Group, Chadstone Centre, VIC, Australia) positioned directly above the OF box by a Manfrotto 190× tripod (JB Hi-Fi Group, Chadstone Centre, VIC, Australia). The apparatus was sanitized with 80% ethanol after each mouse to prevent any olfactory cues that could interfere with animal behaviour. Eztrack software (last updated on the 20 July 2020) was utilized to analyze 3 min of footage per mouse and automatically track the movement of it; this determined the total distance, distance travelled in the outer (safe) zone and inner/centre (unsafe) zone, and time spent in center—a measure of exploratory performance and/or anxiety-related behaviour [72].

### 4.3. Rotarod Test

A RotaRod apparatus (Ugo Basile, Gemonio, Italy, model 47600) with the capacity to test 5 mice concurrently was utilised for this assessment. The apparatus consists of a rotating cylinder separated into 5 segments and a landing plate beneath each that is coupled to a timer. The standard accelerated rotarod test was employed, in which the cylinder was set to accelerate from 4 rotations per minute (RPM) to 40 RPM, over 5 min [73]. The amount of time a mouse successfully remained running on the rotarod (latency) was recorded automatically, as the falling of a mouse onto the plate immediately halted the timer [74]. In the instance a mouse gripped onto the cylinder and revolved with it for a full rotation without engaging in movement (passive turn), the timer was stopped, and the latency recorded. The latency for each animal to fall was assessed over 3 trials and the cylinder and plates cleaned with 80% ethanol between mice [75].

### 4.4. Luxol Fast Blue Staining

Brain and spinal cord tissues were left submerged in paraformaldehyde for 24 h for fixation, then transferred to 70% ethanol for storage. Tissue was then processed (Excelsior™ AS Tissue Processor, Imbros Pty Ltd, Cambridge, Tasmania, Australia) and embedded in paraffin wax (The Epredia™ HistoStar™ embedding workstation). Sagittal and coronal brain sections (striatum and hippocampus regions), in addition to coronal spinal cord sections of 5 μm thickness were cut using a microtome (Epredia™ HM 325 microtome, Thermo Fisher Scientifics, Scoresby, VIC, Australia) and mounted on silane-coated glass slides (StarFrost, ProSciTech, Kirwan, QLD, Australia).

Luxol Fast Blue (LFB) staining began by deparaffinising the sections in xylene (3× for 2 min). Immersion in absolute ethanol (2× for 2 min) followed by 95% ethanol for 2 min then occurred. Slides were placed in LFB solution overnight (for approximately 15 h) in an oven maintained at 57 °C, covered in parafilm to prevent evaporation of the solution. Following this, sections were washed with 95% ethanol, then immersed for a further 2 min before differentiation was carried out by submersion for 30 s in Lithium carbonate for (0.05% in ddH_2_O, 255823-100G, Sigma Aldrich, St. Louis, MO, USA). Sections were then rinsed in distilled water, cleansed in 70% ethanol, rinsed once more returned to distilled water momentarily; this was repeated until tissue structures were adequately distinguishable microscopically and not over-stained.

Next, the tissue was counter stained with 0.1% cresyl violet by immersion for 10 min (C5042-10G, Merck, Rahway, NJ, USA) to identify neurons. Slides were washed in distilled water, then differentiated by submersion for 10 s in 95% ethanol, followed by pure ethanol (2× for 5 min). Lastly, xylene was used (2× for 5 min) and cover slipped with VectaMount Express Mounting medium (H-5700-60, Abacus DX, Cannon Hill, Australia). Microscopic images were acquired on ZEISS AxioScan.Z1 (Carl Zeiss Australasia, NSW, Australia) at ×20 magnification.

### 4.5. Hematoxylin and Eosin (H&E) Staining

Sagittal brain and retinal tissue cross sections were immersed in xylene (3× for 2 min), followed by (2× for 2 min). This was proceeded by 95% ethanol (2× for 2 min), 70% ethanol (1× for 2 min), 50% ethanol (1× for 2 min) and lastly, deionized water (3× for 2 min). Tissue was then immersed in Hematoxylin for 1 min, rinsed thoroughly with water and decolorized by submerging momentarily in 0.5% acid alcohol. Next, slides were washed with 1 min in 1× Phosphate-buffered saline (PBS) followed by three changes of deionized water, and then counterstained with pink Alcoholic-Eosin for 30 s. Lastly, tissue was dehydrated. To do so, tissue was immersed for the same period as before but in increasing concentrations of ethanol, till xylene was reached such that nuclei could be visualized as blue and cytoplasm as pink. Finally, slides were cover slipped with VectaMount Express Mounting medium (H-5700-60, Abacus DX, Cannon Hill, Australia).

### 4.6. Immunofluorescence

Brain sections were introduced into xylene (2× for 10 min) for deparaffinization and then rehydrated in diminishing concentrations of ethanol: pure ethanol (2× for 3 min), 95% ethanol (2× for 3 min), 70% ethanol (1× for 3 min), 50% ethanol (1× for 3 min) and lastly distilled water (1× for 3 min). This was followed by an antigen retrieval step, in which slides were placed in plastic Coplin jars (HL44208-X, ProSciTech, Kirwan, Australia), immersed in sodium citrate buffer (10mM citric acid, 0.05% Tween-20, pH 6.0) and heated with a pressure cooker for 15 min. Slides were cooled for 30 min on ice, prior to tracing around tissue with a hydrophobic marker. Autofluorescence reduction solution (0.25% NH_3_ IN 70% Ethanol) was applied for 1 h. Following this, tissue was permeabilized (1× for 30 min, 0.4% Triton-X100 in PBS) and blocked for 1 h in a humidified chamber to prevent non-specific binding of the antibody (5% BSA composed of 0.2% gelatin, 0.25% Triton-X100 in PBS). Table 2 below details the primary antibodies and titers used for the purpose of this study. Slides were then maintained with primary antibody, dilutions comprising 1% BSA (0.2% gelatin, 0.25% Triton-X100 in PBS) overnight at 4 °C, then incubated with secondary antibody (Anti-Rabbit Alexa-Fluor 488 and/or Anti-Mouse Alexa-Fluor 594, 1:500 dilution in 1% BSA) for 1 h in the dark. Finally, sections were counterstained with DAPI (1:500 in 1× PBS), then cover-slipped with Vectashield^®^ Antifade Mounting Medium.

### 4.7. Image Analysis

Histological and immunohistochemical images were acquired on a ZEISS AxioScan.Z1 slide scanner (Carl Zeiss Australasia, Macquarie Park, NSW, Australia) at ×20 magnification. All analyses were performed using FIJI/ImageJ software (version 1.53k; National Institutes of Health, Bethesda, MD, USA). To ensure unbiased assessment, images were coded and the experimenter blinded prior to analysis.

For myelin quantification, the Color Deconvolution function [76] was applied to separate the blue Luxol Fast Blue (LFB) signal from the purple Cresyl violet counterstain. Consistent thresholding was then applied across all groups, and the extent of myelination was calculated as mean grey intensity within predefined regions of interest (ROIs). ROIs encompassed either the corpus callosum or the motor cortex, which are areas commonly examined in experimental models of demyelination and remyelination [77].

For quantification of the endoplasmic reticulum (ER) stress marker phospho-IRE1 in ASPA^+^ oligodendrocytes within the corpus callosum, the Particle Analysis function in ImageJ was employed. Individual pIRE1^+^/ASPA^+^ cells were identified and counted, and results normalized to ROI area (mm^2^) using the following formula, as previously described by our group [33]:

$$Cells/mm^{2}=\frac{\left(average\;number\;of\;cells\;in\;each\;ROI\times 10^{6}\right)}{ROI\;area(\mu m^{2})}\;$$

To determine the percentage of ER stress-positive neurons in the motor cortex, at least three ROIs per section were analyzed (200 μm × 200 μm = 40,000 μm^2^). TUJ1^+^ neurons double-positive for pIRE1 were quantified relative to the total TUJ1^+^ neuronal population within each ROI, according to the formula:

$$\%pIRE^{+}neurons=\frac{\left(TUJ1^{+}pIRE1^{+}positive\;neurons\;per\;ROI\right)}{\left(total\;TUJ1^{+}neurons\;per\;ROI\right)}\times 100$$

This multiparametric approach to image analysis is widely used for quantitative histology in neurodegenerative and demyelinating disease models [77,78].

### 4.8. Immunohistochemistry

Spinal cord cross-sections, obtained at the same spinal depth as for LFB staining were immersed in xylene, followed by declining concentrations of ethanol as described in Section 2.5. To detect Synapsin II (1:500, GTX135310, GeneTex, Irvine, CA, USA), tissue sections were incubated overnight at 4 °C in a humidified chamber using Rabbit-Specific HRP/DAB Detection IHC Kit (ab64261, Abcam, Cambridge, UK). This was followed by counterstaining with Hematoxylin to visualize cell nuclei. Sections were exposed to rising concentrations of ethanol, and lastly xylene before cover slipping via VectaMount Express Mounting medium (Abacus DX, Cannon Hill, Australia). Images were obtained using ZEISS AxioScan.Z1 (Carl Zeiss Australasia, Macquarie Park, NSW, Australia) at 20× magnification.

### 4.9. Real-Time Quantitative Polymerase Chain Reaction (qPCR)

Brains were collected by carefully snipping along the sagittal midline and lateral surfaces of the mouse cranium with surgical scissors. Brain and spinal tissue were immersed in RNase inhibitor solution then viewed through a dissecting microscope (10× magnification), upon an ice-cold black surface for elevated contrast. The brain was then incised coronally at approximately +0.5 to +1.0 mm from Bregma. Following this, white matter tissue comprising the corpus callosum situated above the striatum was isolated, by making an excision at −1.0 to −1.5 mm. These same steps were implemented for isolation of the motor cortex, with an incision at +1.10 to +1.20 mm from Bregma, and −0.5 to −1.0 mm, respectively. Two 23-gauge needles were then employed to separate any cortical tissue from white matter isolates, and vice versa for motor cortex isolates; these were then immersed immediately in liquid nitrogen until further experimentation. TRI-reagent (T9424-200ML, Sigma Aldrich) was therein utilised to extract RNA from dissected tissue, and 2-proponal employed for formation of an RNA precipitate, as detailed in prior literature [79]. Following this, complementary DNA (cDNA) was generated from RNA using the Tetro cDNA Synthesis kit (BIO-65043, Bioline, Memphis, TN, USA), and real time (RT)-qPCRs was then conducted to quantify the gene expression of relevant myelin and ER stress markers in the white matter, motor cortex and hippocampal regions of the brain, in addition to the spinal cord. Each RT-qPCR reaction consisted of 5 µL of iTaq Universal SYBR Green Supermix (Cat. No. 1725124, Bio-Rad, Hercules, CA, USA), 3 µL of cDNA (100 ng), and 0.8 µL of forward and reverse primers (final concentration = 400 nM). Ribosomal protein S18 was employed as the housekeeping gene and the fold change calculated to determine expression levels of each gene via the 2^−ΔΔCt^ method, as described prior [80]. Table 3 below details the primers and nucleotide sequences used for this study.

### 4.10. Tissue Protein Extraction and Western Blots

Spinal cord tissues were homogenized in radioimmunoprecipitation assay (RIPA) buffer using a sterile autoclavable pestle. Buffer volumes were calculated at a weight to volume ratio of 1:10, and protease inhibitors were added to prevent protein degradation.

Samples were sonicated three times (10 s bursts at 50% intensity), with 30 s cooling on ice between bursts. The homogenate was centrifuged at 12,000× *g* for 10 min at 4 °C. The supernatant was collected and stored at −80 °C until use.

For protein denaturation, 30 µg of extracted protein was mixed with 3.75 µL of Laemmli buffer and β-mercaptoethanol (9:1 ratio). The mixture was vortexed, centrifuged, and incubated at 70 °C for 10 min in a T100™ thermal cycler (Bio-Rad).

Proteins were separated on a 4–20% Mini-PROTEAN^®^ TGX Stain-Free™ protein gel (Bio-Rad) using sodium dodecyl sulfate-polyacrylamide gel electrophoresis (SDS-PAGE). Resolved proteins were then transferred to a PVDF membrane, followed by incubation for 1 h in blocking buffer composed of 5% skimmed milk in 1× TBST. Primary antibodies utilized for Western blots and the respective dilutions are detailed in Table 4 below. Incubation in primary antibody was conducted with overnight oscillations at 4 °C. Lastly, bands were visualized with Clarity™ Western ECL Blotting Substrate (Bio-Rad) and imaged via the ChemiDoc MP System (Bio-Rad, Gladesville, NSW, Australia).

### 4.11. Statistical Analyses

All statistical analyses were conducted on GraphPad prism (version 9.3.1) and results are presented as the mean ± SEM, unless otherwise indicated. One-way ANOVA was utilized to compare differences among genotypes in RT-qPCRs and imaging studies, while mixed-effects linear models were employed for all behavioral analyses. *p*-values ≤ 0.05 were considered statistically significant.

## Figures and Tables

**Table 1:** Summary of results.

Type of Investigation	PAC1^+/−^ Mice (vs. PAC1^+/+^ Mice)	PAC1^−/−^ Mice (vs. PAC1^+/+^ Mice)	CNS Area
Animal Phenotypes			
Pre-weaning survivability	Unchanged	↓ Increased	n/a
Animal weight	Unchanged	↓ Decreased	n/a
Rotarod latency (Week 0)	Unchanged	↓ Decreased	n/a
Open Field Test—total distance travelled (Week 0)	Unchanged	↑ Increased	n/a
Open Field Test—time spent in centre (Week 0)	Unchanged	↑ Increased	n/a
Gene Expression Studies			
PLP1	Unchanged	↓ Decreased	White Matter
Olig2	↓ Decreased	↓ Decreased	White Matter
MBP	↓ Decreased	↓ Decreased	Motor Cortex
Olig2	↑ Increased	↑ Increased	Motor Cortex
PERK	Unchanged	↑ Increased	Motor Cortex
ERN1	↓ Decreased	↓ Decreased	Motor Cortex
Histology/IF			
Luxol Fast Blue (Myelin Density)	Unchanged	↓ Decreased	Corpus Callosum, Cortex and Hippocampus
ASPA	Unchanged	↓ Decreased	Corpus Callosum
pIRE1	Unchanged	↑ Increased	Motor Cortex
Retinal thickness	Unchanged	↓ Decreased	Retina
GCL thickness	Unchanged	↓ Decreased	Retina

**Table 2:** Primary antibodies used to examine myelin and ER stress markers via immunofluorescence in this study.

Antibody	Species	Dilution	Product Code
Recombinant Monoclonal ASPA antibody	Rabbit	1:500	Abcam, ab223269, EPR22072
β-III tubulin (TUJ1) Monoclonal Antibody	Mouse	1:500	Bio Legend, Cat# 80120, San Diego, CA, USA
Phosphorylated Inositol-Requiring Enzyme 1 (pIRE1) Antibody (Ser 724)	Rabbit	1:250	Sigma Aldrich, Cat# ZRB1072-25UL

**Table 3:** The qPCR primers and their respective nucleotide sequences employed to examine myelin and ER stress markers.

Gene	Gene Bank Accession Number	Primer Sequence	Length (bp)
*S18*	NM_011296.2	Fwd: CCCTGAGAAGTTCCAGCACA Rev: GGTGAGGTCGATGTCTGCTT	145
*MOG*	NM_010814.2	Fwd: CTTCTTCAGAGACCACTCTTACC Rev: CCCAATAGAAGGGATCTTCCAC	71
*MBP*	NM_001025251.2	Fwd: TATAAATCGGCTCACAAGGGATT Rev: TGTCTCTTCCTCCCAGCTTA	85
*Olig2*	NM_016967.2	Fwd: AAAGACAAGAAGCAGATGACTGA Rev: AGCATGAGGATGTAGTTTCGC	200
*PLP1*	NM_011123.4	Fwd: ATGCCAGAATGTATGGTGTTCT Rev: TTTAAGGACGGCGAAGTTGTAAG	200
*ERN1*	NM_023913.2	Fwd: GAGACAAAGGAGAGTGTGTGAT Rev: TCAAGTAGTTCAGCTTGCTCTT	87
*PERK*	NM_010121.3	Fwd: CCTTGGTTTCATCTAGCCTCA Rev: ACTTGTAGGAAGATTCGAGCAG	156
*DDIT3 (CHOP)*	NM_007837.4	Fwd: GCTCTCCAGATTCCAGTCAG Rev: CTCCTTCTCCTTCATGCGTT	131
*ATF4*	NM_009716.3	Fwd: CCTCAGACAGTGAACCCAAT Rev: AATGCTCTGGAGTGGAAGAC	127
*ATF6*	NM_001081304.1	Fwd: GAGCTGTCTGTGTGATGATAGT Rev: CTAGGTTTCACTCTTCGGGATT	94

**Table 4:** Primary antibodies employed to examine myelin and synaptic markers in the spinal cord.

Antibody	Dilution	Product Code
Myelin Basic Protein (MBP)	1:1000	GTX133108, GeneTex
Synaptophysin	1:1000	MA514532, ThermoFisher
Synapsin II	1:500	GTX135310, GeneTex
GAPDH	1:2000	VPA00187, BioRad

## Data Availability

All data are reported in the published version of this article. Raw data can be made available upon reasonable request to the authors.
